# *N*-Crotylphthalimide

**DOI:** 10.1107/S1600536810046039

**Published:** 2010-11-17

**Authors:** Marcos Flores-Alamo, María del Carmen Romero-Quiroz, Jorge Morgado

**Affiliations:** a Facultad de Química, Universidad Nacional Autónoma de México, Coyoacán 04360, DF, Mexico

## Abstract

In the title compound {systematic name: 2-[(*E*)-but-2-en-1-yl]isoindoline-1,3-dione}, C_12_H_11_NO_2_, the phthalimide ring system is essentially planar, with a maximum deviation of 0.008 (1) Å, while the plane of the *N*-crotyl substituent is orthogonal to the phthalimide ring system, making a dihedral angle of 87.5 (1)°.

## Related literature

For related structures, see: Warzecha, Görner & Griesbeck (2006 ▶): Warzecha, Lex & Griesbeck (2006 ▶); Mustaphi *et al.* (2001 ▶). For details of inter­molecular inter­actions, see: Desiraju (1991 ▶); Steiner (2002 ▶); Etter *et al.* (1990 ▶). For the synthesis of *N*-crotylphtalimide, see: Roberts & Mazur (1951 ▶); Mowery *et al.* (2007 ▶). 
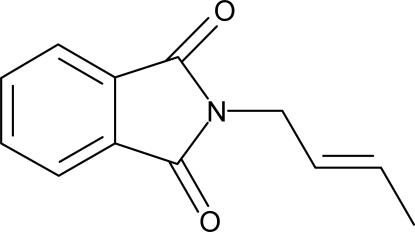


## Experimental

### 

#### Crystal data

C_12_H_11_NO_2_
*M* *_r_* = 201.22
Monoclinic, 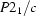

*a* = 8.1880 (4) Å
*b* = 12.0830 (5) Å
*c* = 10.8080 (5) Å
β = 110.431 (4)°
*V* = 1002.03 (8) Å^3^
*Z* = 4
Mo *K*α radiation
μ = 0.09 mm^−1^
*T* = 123 K
0.36 × 0.32 × 0.2 mm

#### Data collection

Oxford Diffraction Gemini Atlas CCD diffractometer
Absorption correction: analytical (*CrysAlis RED*; Oxford Diffraction, 2009 ▶) *T*_min_ = 0.973, *T*_max_ = 0.983
7022 measured reflections
1959 independent reflections
1669 reflections with *I* > 2σ(*I*)
*R*_int_ = 0.016

#### Refinement

*R*[*F* ^2^ > 2σ(*F* ^2^)] = 0.032
*wR*(*F* ^2^) = 0.080
*S* = 1.06
1959 reflections
137 parameters
H-atom parameters constrained
Δρ_max_ = 0.20 e Å^−3^
Δρ_min_ = −0.19 e Å^−3^

### 

Data collection: *CrysAlis CCD* (Oxford Diffraction (2009 ▶); cell refinement: *CrysAlis RED* (Oxford Diffraction 2009 ▶); data reduction: *CrysAlis RED*; program(s) used to solve structure: *SHELXS97* (Sheldrick, 2008 ▶); program(s) used to refine structure: *SHELXL97* (Sheldrick, 2008 ▶); molecular graphics: *ORTEP-3* (Farrugia, 1997 ▶); software used to prepare material for publication: *WinGX* (Farrugia, 1999 ▶).

## Supplementary Material

Crystal structure: contains datablocks I, global. DOI: 10.1107/S1600536810046039/is2623sup1.cif

Structure factors: contains datablocks I. DOI: 10.1107/S1600536810046039/is2623Isup2.hkl

Additional supplementary materials:  crystallographic information; 3D view; checkCIF report

## Figures and Tables

**Fig. 1.**
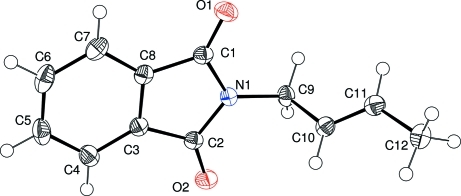
The molecular structure and the atom-labelling scheme for (I). Displacement ellipsoids are drawn at the 50% probability level and H atoms are shown as circles of arbitrary size.

**Fig. 2.**
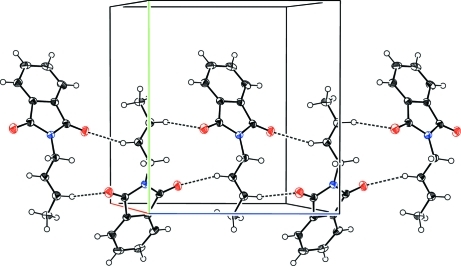
Intermolecular contacts of van der Waals (dashed lines) in the crystal structure of (I), forming cross-linked ribbons of R22 (13) motifs.

## supplementary crystallographic information

### Comment

Structure-activity relationship studies on *N*-allylphthalimide derivatives indicated that the influence of the allylic substituent on the photophysical and electrochemical properties of the phthalimide chromophore, which plays an important role as an electron acceptor in photo-induced electron-transfer reactions (Warzecha, Görner & Griesbeck, 2006).

The structure of *N*-crotylphthalimide (I) is shown in Fig. 1. The 2-butenyl substituent at the imide N atom of the planar phthalimide adopts an antiperiplanar conformation with torsion angles C1—N1—C9—C10 and N1—C9—C10—C11 of 92.53 and -131.59 (1)°; to difference of the orientation synperiplanar of *N*-Allylphthalimide (Mustaphi *et al.*, 2001; Warzecha, Lex & Griesbeck, 2006). The phthalimide ring system is essentially planar, with a maximum deviation of 0.008 (1)Å for atom C1 and dihedral angle of 0.13 to 1.39 (1)°, for other hand, the dihedral angle of 92.53 (1)° evidence that the *N*-crotyl group is orthogonal to the the phthalimide ring plane.

In the crystal structure, the intermolecular contact of 2.649 (1) Å between each molecule features pairs of C_vinyl_—H and O=C bonds (Desiraju, 1991; Steiner, 2002) to its neighbours related by the symmetry operations *x*,-*y* + 1/2,+*z* + 1/2 and *x*,-*y* + 1/2,+*z* - 1/2 mainly. These interactions of van der Waals lead to infinite ribbons of *R*^2^_2_ (13) motifs (Etter & MacDonald, 1990), as illustrated in Fig. 2. The ribbons run in the direction of the crystallographic *c* axis.

### Experimental

*N*-crotylphtalimide (I) was prepared according to the procedure reported (Roberts & Mazur, 1951). Under argon, crotyl bromide (85% pure; 25 g; 157.4 mmol) was added with stirring at room temperature to a white suspension of potassium phthalimide (43.7 g; 236 mmol) in dimethylformamide (150 ml). The mixture was heated at 120° C for 30 minutes and then at 160° C for an additional 30 minutes. The hot mixture was poured over 50 g of ice-water and extracted with chloroform (4 × 20 ml). The combined extracts were washed successively with 1 N NaOH, water, 0.5 N HCl and again with water. The chloroform solution was dried over MgSO_4_ and filtered.

### Refinement

H atoms attached to C atoms were placed in geometrically idealized positions and refined as riding on their parent atoms, with C—H = 0.93–0.97 Å, and with *U*_iso_(H) = 1.2*U*_eq_(C) or *U*_iso_(H) = 1.5*U*_eq_(C) for methyl groups.

### Crystal data

**Table:**

C_12_H_11_NO_2_	*F*(000) = 424
*M**_r_* = 201.22	*D*_x_ = 1.334 Mg m^−^^3^
Monoclinic, *P*2_1_/*c*	Mo *K*α radiation, λ = 0.71073 Å
*a* = 8.1880 (4) Å	Cell parameters from 4695 reflections
*b* = 12.0830 (5) Å	θ = 3.8–26.0°
*c* = 10.8080 (5) Å	µ = 0.09 mm^−^^1^
β = 110.431 (4)°	*T* = 123 K
*V* = 1002.03 (8) Å^3^	Block, colorless
*Z* = 4	0.36 × 0.32 × 0.2 mm

### Data collection

**Table:**

Oxford Diffraction Gemini Atlas CCD diffractometer	1959 independent reflections
graphite	1669 reflections with *I* > 2σ(*I*)
Detector resolution: 10.4685 pixels mm^-1^	*R*_int_ = 0.016
ω scans	θ_max_ = 26.1°, θ_min_ = 3.9°
Absorption correction: analytical (*CrysAlis RED*; Oxford Diffraction, 2009)	*h* = −10→10
*T*_min_ = 0.973, *T*_max_ = 0.983	*k* = −14→13
7022 measured reflections	*l* = −13→10

### Refinement

**Table:**

Refinement on *F*^2^	Primary atom site location: structure-invariant direct methods
Least-squares matrix: full	Secondary atom site location: difference Fourier map
*R*[*F*^2^ > 2σ(*F*^2^)] = 0.032	Hydrogen site location: inferred from neighbouring sites
*wR*(*F*^2^) = 0.080	H-atom parameters constrained
*S* = 1.06	*w* = 1/[σ^2^(*F*_o_^2^) + (0.0389*P*)^2^ + 0.204*P*] where *P* = (*F*_o_^2^ + 2*F*_c_^2^)/3
1959 reflections	(Δ/σ)_max_ < 0.001
137 parameters	Δρ_max_ = 0.20 e Å^−^^3^
0 restraints	Δρ_min_ = −0.19 e Å^−^^3^

### Special details

**Table:**

Geometry. All s.u.'s (except the s.u. in the dihedral angle between two l.s. planes) are estimated using the full covariance matrix. The cell s.u.'s are taken into account individually in the estimation of s.u.'s in distances, angles and torsion angles; correlations between s.u.'s in cell parameters are only used when they are defined by crystal symmetry. An approximate (isotropic) treatment of cell s.u.'s is used for estimating s.u.'s involving l.s. planes.
Refinement. Refinement of *F*^2^ against ALL reflections. The weighted *R*-factor *wR* and goodness of fit *S* are based on *F*^2^, conventional *R*-factors *R* are based on *F*, with *F* set to zero for negative *F*^2^. The threshold expression of *F*^2^ > 2σ(*F*^2^) is used only for calculating *R*-factors(gt) *etc*. and is not relevant to the choice of reflections for refinement. *R*-factors based on *F*^2^ are statistically about twice as large as those based on *F*, and *R*- factors based on ALL data will be even larger.

### Fractional atomic coordinates and isotropic or equivalent isotropic displacement parameters (Å^2^)

**Table:**

	*x*	*y*	*z*	*U*_iso_*/*U*_eq_	
O1	0.32549 (11)	0.11878 (7)	0.22557 (8)	0.0293 (2)	
O2	−0.00316 (11)	0.09982 (7)	−0.21251 (8)	0.0299 (2)	
N1	0.13954 (11)	0.13240 (7)	0.00921 (9)	0.0201 (2)	
C3	0.21517 (14)	−0.02776 (9)	−0.07262 (11)	0.0200 (2)	
C1	0.26809 (13)	0.08188 (9)	0.11487 (10)	0.0202 (2)	
C8	0.31372 (14)	−0.02229 (9)	0.06096 (11)	0.0200 (2)	
C7	0.43062 (14)	−0.10496 (10)	0.12205 (12)	0.0260 (3)	
H7	0.4963	−0.1017	0.2116	0.031*	
C11	0.19618 (14)	0.42523 (9)	0.04805 (11)	0.0237 (3)	
H11	0.175	0.4284	0.1271	0.028*	
C2	0.10193 (14)	0.07222 (9)	−0.10729 (10)	0.0204 (2)	
C12	0.27941 (15)	0.52388 (9)	0.01158 (12)	0.0276 (3)	
H12A	0.303	0.5085	−0.0677	0.041*	
H12B	0.3866	0.5405	0.0819	0.041*	
H12C	0.2022	0.5862	−0.0029	0.041*	
C4	0.23032 (15)	−0.11498 (9)	−0.15043 (12)	0.0259 (3)	
H4	0.1643	−0.1181	−0.24	0.031*	
C9	0.05901 (14)	0.23939 (9)	0.01554 (11)	0.0229 (3)	
H9A	0.0605	0.2512	0.1047	0.027*	
H9B	−0.0617	0.2379	−0.0429	0.027*	
C6	0.44640 (15)	−0.19339 (10)	0.04444 (13)	0.0307 (3)	
H6	0.5239	−0.2503	0.0829	0.037*	
C5	0.34873 (16)	−0.19817 (10)	−0.08913 (13)	0.0307 (3)	
H5	0.3625	−0.258	−0.1387	0.037*	
C10	0.15001 (14)	0.33373 (9)	−0.02266 (11)	0.0217 (3)	
H10	0.1753	0.3277	−0.0999	0.026*	

### Atomic displacement parameters (Å^2^)

**Table:**

	*U*^11^	*U*^22^	*U*^33^	*U*^12^	*U*^13^	*U*^23^
O1	0.0324 (5)	0.0331 (5)	0.0201 (4)	−0.0027 (4)	0.0062 (4)	−0.0045 (3)
O2	0.0310 (5)	0.0331 (5)	0.0208 (4)	0.0034 (4)	0.0031 (4)	0.0009 (3)
N1	0.0217 (5)	0.0184 (5)	0.0202 (5)	0.0006 (4)	0.0073 (4)	−0.0009 (4)
C3	0.0203 (5)	0.0184 (5)	0.0235 (6)	−0.0041 (4)	0.0106 (4)	0.0005 (4)
C1	0.0197 (5)	0.0218 (6)	0.0201 (6)	−0.0037 (4)	0.0081 (5)	0.0013 (4)
C8	0.0188 (5)	0.0194 (6)	0.0239 (6)	−0.0039 (4)	0.0099 (4)	0.0015 (4)
C7	0.0210 (6)	0.0256 (6)	0.0312 (6)	−0.0014 (5)	0.0088 (5)	0.0071 (5)
C11	0.0219 (6)	0.0254 (6)	0.0243 (6)	0.0029 (5)	0.0084 (5)	0.0019 (5)
C2	0.0199 (5)	0.0215 (6)	0.0204 (6)	−0.0036 (4)	0.0079 (5)	0.0001 (4)
C12	0.0249 (6)	0.0228 (6)	0.0335 (7)	0.0003 (5)	0.0083 (5)	0.0007 (5)
C4	0.0304 (6)	0.0220 (6)	0.0293 (6)	−0.0064 (5)	0.0154 (5)	−0.0039 (5)
C9	0.0228 (6)	0.0199 (6)	0.0278 (6)	0.0023 (4)	0.0112 (5)	−0.0013 (4)
C6	0.0254 (6)	0.0206 (6)	0.0503 (8)	0.0025 (5)	0.0185 (6)	0.0080 (5)
C5	0.0360 (7)	0.0181 (6)	0.0470 (8)	−0.0027 (5)	0.0259 (6)	−0.0035 (5)
C10	0.0202 (5)	0.0219 (6)	0.0242 (6)	0.0033 (4)	0.0093 (5)	0.0021 (4)

### Geometric parameters (Å, °)

**Table:**

O1—C1	1.2076 (13)	C11—H11	0.93
O2—C2	1.2091 (13)	C12—H12A	0.96
N1—C2	1.3923 (14)	C12—H12B	0.96
N1—C1	1.3948 (14)	C12—H12C	0.96
N1—C9	1.4637 (14)	C4—C5	1.3931 (17)
C3—C4	1.3807 (15)	C4—H4	0.93
C3—C8	1.3880 (16)	C9—C10	1.4966 (15)
C3—C2	1.4888 (15)	C9—H9A	0.97
C1—C8	1.4885 (15)	C9—H9B	0.97
C8—C7	1.3815 (16)	C6—C5	1.3860 (18)
C7—C6	1.3920 (17)	C6—H6	0.93
C7—H7	0.93	C5—H5	0.93
C11—C10	1.3220 (16)	C10—H10	0.93
C11—C12	1.4926 (16)		
			
C2—N1—C1	112.17 (9)	H12A—C12—H12B	109.5
C2—N1—C9	122.86 (9)	C11—C12—H12C	109.5
C1—N1—C9	124.88 (9)	H12A—C12—H12C	109.5
C4—C3—C8	121.78 (10)	H12B—C12—H12C	109.5
C4—C3—C2	130.28 (10)	C3—C4—C5	117.17 (11)
C8—C3—C2	107.94 (9)	C3—C4—H4	121.4
O1—C1—N1	124.83 (10)	C5—C4—H4	121.4
O1—C1—C8	129.49 (10)	N1—C9—C10	112.59 (9)
N1—C1—C8	105.68 (9)	N1—C9—H9A	109.1
C7—C8—C3	121.13 (10)	C10—C9—H9A	109.1
C7—C8—C1	130.62 (10)	N1—C9—H9B	109.1
C3—C8—C1	108.25 (9)	C10—C9—H9B	109.1
C8—C7—C6	117.49 (11)	H9A—C9—H9B	107.8
C8—C7—H7	121.3	C5—C6—C7	121.23 (11)
C6—C7—H7	121.3	C5—C6—H6	119.4
C10—C11—C12	125.45 (11)	C7—C6—H6	119.4
C10—C11—H11	117.3	C6—C5—C4	121.20 (11)
C12—C11—H11	117.3	C6—C5—H5	119.4
O2—C2—N1	124.52 (10)	C4—C5—H5	119.4
O2—C2—C3	129.55 (10)	C11—C10—C9	123.16 (10)
N1—C2—C3	105.93 (9)	C11—C10—H10	118.4
C11—C12—H12A	109.5	C9—C10—H10	118.4
C11—C12—H12B	109.5		
			
C2—N1—C1—O1	178.65 (10)	C1—N1—C2—C3	1.06 (12)
C9—N1—C1—O1	2.11 (17)	C9—N1—C2—C3	177.68 (9)
C2—N1—C1—C8	−1.51 (12)	C4—C3—C2—O2	−0.1 (2)
C9—N1—C1—C8	−178.05 (9)	C8—C3—C2—O2	179.95 (11)
C4—C3—C8—C7	−0.57 (16)	C4—C3—C2—N1	179.84 (11)
C2—C3—C8—C7	179.40 (9)	C8—C3—C2—N1	−0.13 (11)
C4—C3—C8—C1	179.26 (10)	C8—C3—C4—C5	0.25 (16)
C2—C3—C8—C1	−0.77 (11)	C2—C3—C4—C5	−179.71 (10)
O1—C1—C8—C7	1.02 (19)	C2—N1—C9—C10	−83.65 (12)
N1—C1—C8—C7	−178.81 (11)	C1—N1—C9—C10	92.53 (12)
O1—C1—C8—C3	−178.78 (11)	C8—C7—C6—C5	0.12 (17)
N1—C1—C8—C3	1.39 (11)	C7—C6—C5—C4	−0.43 (17)
C3—C8—C7—C6	0.37 (16)	C3—C4—C5—C6	0.24 (16)
C1—C8—C7—C6	−179.42 (10)	C12—C11—C10—C9	−176.94 (10)
C1—N1—C2—O2	−179.01 (10)	N1—C9—C10—C11	−131.59 (11)
C9—N1—C2—O2	−2.39 (16)		
